# Supplementary material for: Image-based screen capturing misfolding status of Niemann-Pick type C1 identifies potential candidates for chaperone drugs
Source: PLoS One. 2020 Dec 14;15(12):e0243746. doi: 10.1371/journal.pone.0243746 (PMC7735562; doi:10.1371/journal.pone.0243746)
Supplement: S1 Appendix — (PDF) [file pone.0243746.s001.pdf]

Supplementary Appendix S1 for:

Image-based screen capturing misfolding status of Niemann-Pick type C1 identifies potential chaperone drugs

Ryuta Shioi[1], Fumika Karaki[1,#a], Hiromasa Yoshioka[1], Tomomi Noguchi-Yachide[1], Minoru Ishikawa[2], Kosuke Dodo[3], Yuichi Hashimoto[1], Mikiko Sodeoka[3], and Kenji Ohgane[1,3,#b*]

[1] Institute for Quantitative Biosciences, the University of Tokyo, 1-1-1 Yayoi, Bunkyo-ku, Tokyo 113-0032, Japan

[2] Graduate School of Life Sciences, Tohoku University, 2-1-1 Katahira, Aoba-ku, Sendai 980-8577, Japan

[3] Synthetic Organic Chemistry Laboratory, RIKEN Cluster for Pioneering Research, 2-1 Hirosawa, Wako, Saitama 351-0198, Japan

[#a] Current address: Laboratory of Medicinal Chemistry, School of Pharmacy, Kitasato University, 5-9-1, Shirokane, Minato-ku, Tokyo 108-8641, Japan

[#b] Current address: Department of Applied Bioscience, Faculty of Science and Technology, Tokyo University of Science, 2645, Yamazaki, Noda, Chiba 278-8510, Japan

* Corresponding author (E-mail: ohgane@rs.tus.ac.jp)

Table of Contents

Supplementary Schemes **3**

Scheme S1. Synthesis of the left-half unit of itraAZY 3

Scheme S2. Synthesis of itraAZY 4

Scheme S3. Synthesis of itraACT 5

Scheme S4. Synthesis of itraBIO 6

Scheme S5. Synthesis of lapaAZY 6

Supplementary Materials and Methods **7**

Abbreviations 7

General methods for organic syntheses 7

Synthesis of itraAZY (Scheme S1-2). 7

Synthesis of itraACT (Scheme S3) 12

Synthesis of itraBIO (Scheme S4) 15

Synthesis of lapaAZY (Scheme S5) 17

Supplementary References **19**

Supplementary Schemes

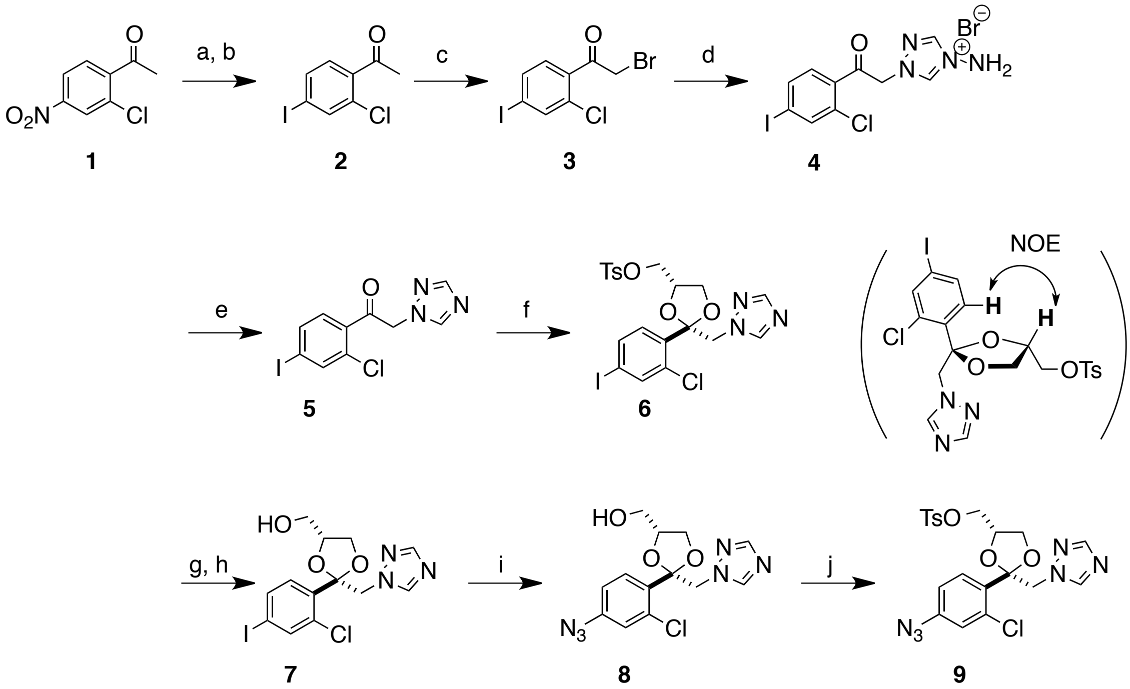

Scheme S1. Synthesis of the left-half unit of itraAZY

Reagents and conditions: (a) Fe, NH_4Cl, $MeOH/H_2O$, reflux, 1 h; (b) $NaNO_2$, aqueous 6M H_2SO_4, aqueous KI, r.t., 10 h (78% over 2 steps); (c) NBS, PTSA, MeCN, 60 ºC, 19 h; (d) 4-amino-1,2,4-triazole, IPA, reflux, 1.5 h (49% over 2 steps); (e) $NaNO_2$, HCl aq. from 0 ºC to r.t., 1 h, (quant.); (f) 1-Tosylglycerol, TfOH, toluene, r.t., 3 day (44%); (g) NaOAc, DMF, reflux, 17 h; (h) Cs_2CO_3, MeOH (quant. over 2 steps); (i) NaN_3, trans-N,N'-dimethylcyclohexane-1,2-diamine, CuI, L-sodium ascorbate, $EtOH/H_2O$, reflux, 3 h (82%); (j) TsCl, Et_3N, DCE, 60 ºC, 20 h (quant.).

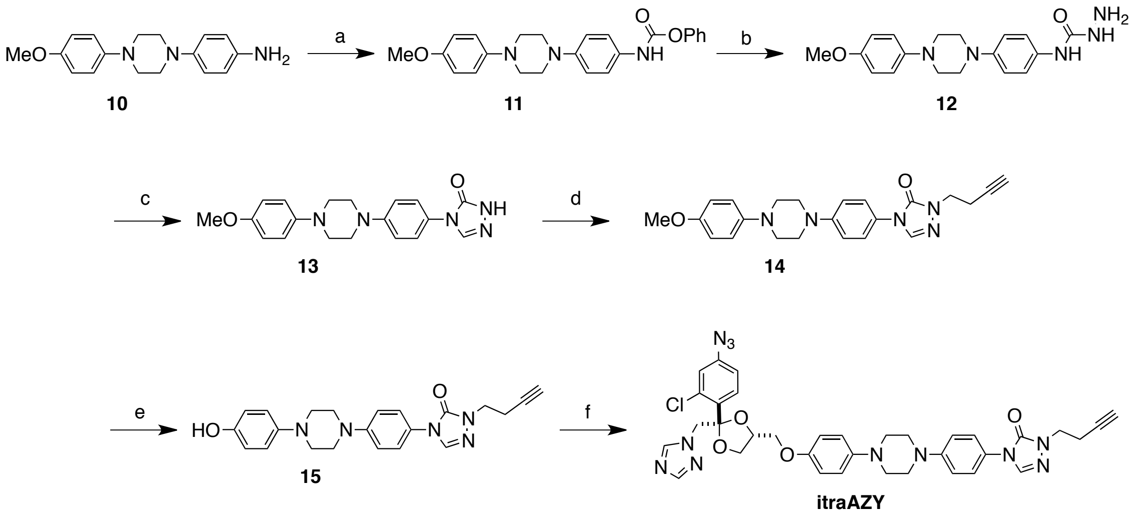

Scheme S2. Synthesis of itraAZY

Reagents and conditions: (a) phenylchloroformate, pyridine, $CHCl_3$, r.t., 1 h; (b) $NH_2NH_2 \cdot H_2O$, 1, 4-dioxane, reflux, 3.5 h; (c) formamidineacetate, *i*PrOH/DMF (1:1), 150 °C, 8 h (76% over 3 steps); (d) 4-bromo-1-butyne, Cs_2CO_3, DMF 100 °C, 24 h, (67%); (e) BBr_3, DCM, r.t., 16 h (66%); (f) **9**, Cs_2CO_3, DMF, 90°C, 2 h (25%).

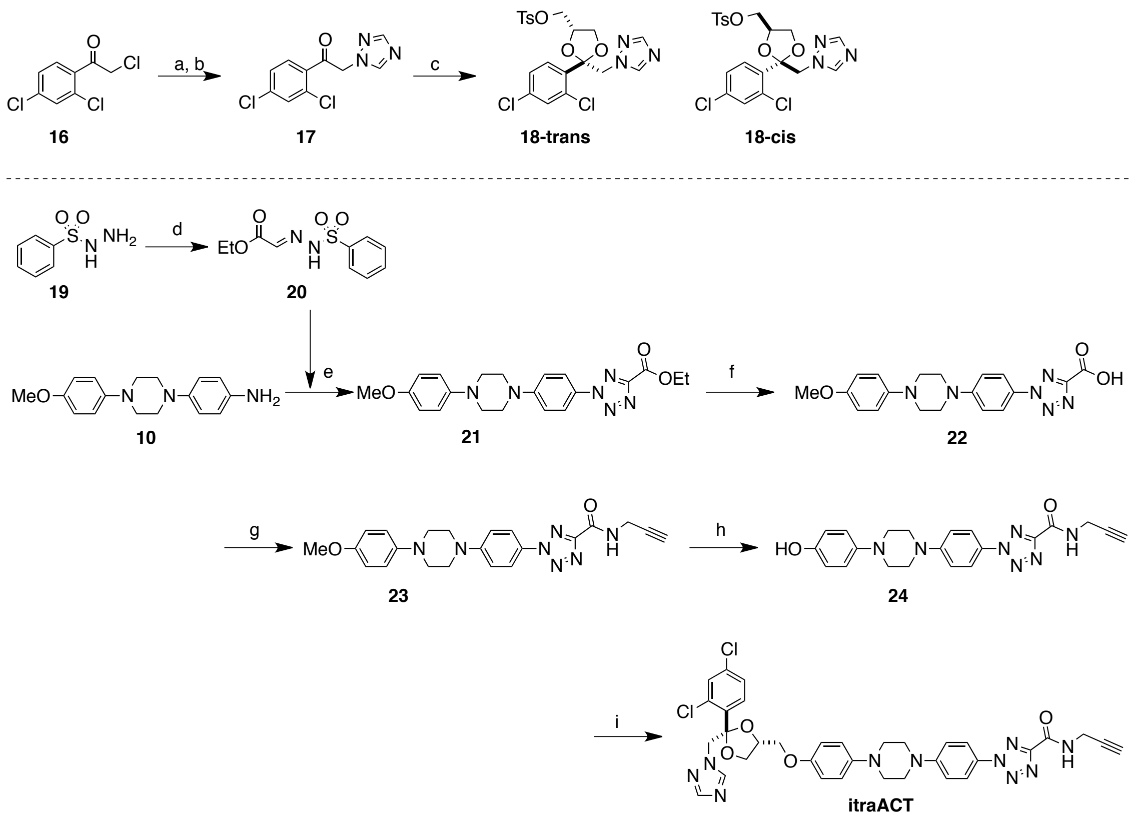

Scheme S3. Synthesis of itraACT

Reagents and conditions: (a) 4-amino-1,2,4-triazole, IPA, refluxed, 10 h; (b) $NaNO_2$, HCl, H_2O, r.t., 3.5 h (63% over 2 steps); (c) 1-tosylglycerol, TfOH, toluene, r.t., 3 days (22%) (the other diastereomer 5%); (d) Ethyl glyoxlate polymer form, EtOH r.t., 1 h; (e) $NaNO_2$, c.HCl, **20** in pyridine, EtOH/H_2O, r.t., 2 h (29%); (f) 5 N NaOH, THF/EtOH, r.t., 16 h; (g) propargylamine, HATU, DIPEA, DMF, 90 °C, 2 h (60% over 2 steps); (h) BBr_3, DCM, r.t., 20 h (95%); (i) **18-trans**, Cs_2CO_3, DMF, 90 °C, 2 h (57%).

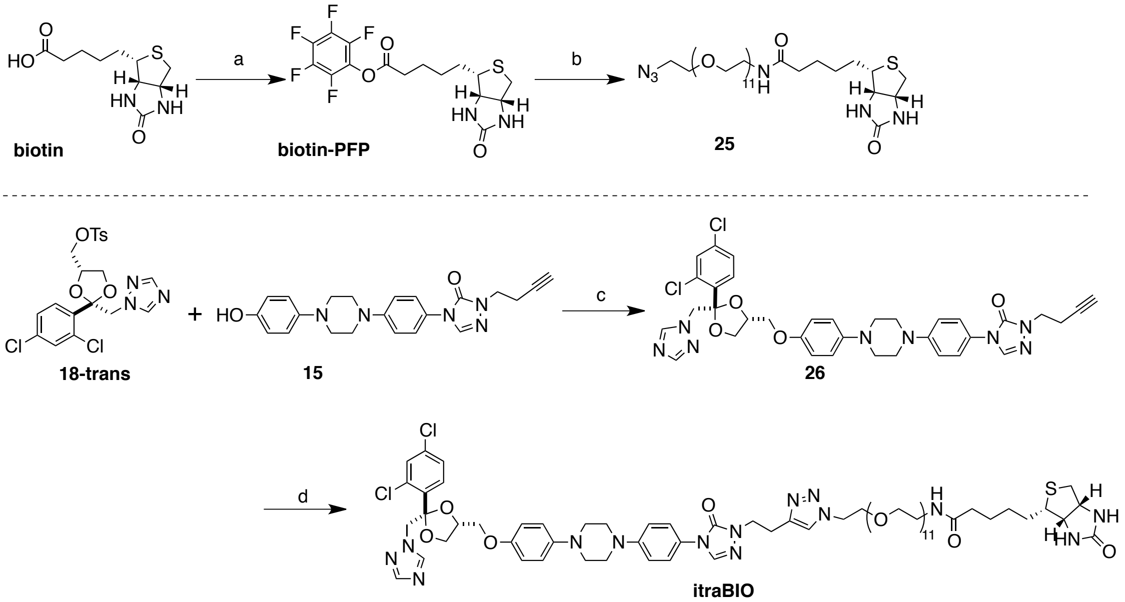

Scheme S4. Synthesis of itraBIO

Reagents and conditions: (a) pentafluorophenyl trifluoroacetate, Et_3N, DMF, reflux, 1 h; (b) azido-PEG_{11}-amine, Et_3N, THF, r.t., 24 h (82%); (c) **18-trans**, Cs_2CO_3, DMF, 100 °C, 5 h (68%); (d) DIPEA, CuI, MeCN, r.t., 5 day (49%).

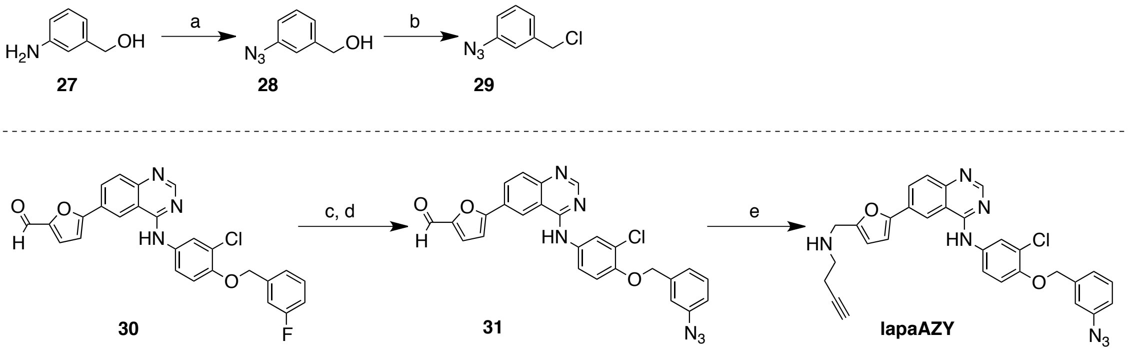

Scheme S5. Synthesis of lapaAZY

Reagents and conditions: (a) $NaNO_2$, NaN_3, 6M HCl, r.t., 30 min (quant.); (b) TsCl, pyridine, DCM, r.t., 15 h (40%); (c) BBr_3, DCM, r.t., 1.5 h; (d) **29**, K_2CO_3, DMF, 80 °C, 1 h (19% over 2 steps); (e) 3-buthynylamine, AcOH, $NaBH(OAc)_3$, 1,2-dichloroethane, r.t., 1 h (64%).

Supplementary Materials and Methods

Abbreviations

Following abbreviations were used: calcd (calculated), DCE (1,2-dichloroethane), DCM (dichloromethane), DIPEA (N,N-diisopropylethylamine), DMF (N,N-dimethylformamide), DMSO (dimethylsulfoxide), ESI (electrospray ionization), HATU (hexafulrophosphate azabenzotriazole tetramethyl uronium), HMBC (heteronuclear multiple bond correlation), HRMS (high-resolution mass spectrometry), NBS (N-bromosuccinimide), PEG (polyethyleneglycol), PFP (pentafluorophenyl), PTSA (para-toluenesulfonc acid), TfOH (trifluoromethanesulfonic acid), TOF (time-of-flight).

General methods for organic syntheses

All chemical reagents and solvents were purchased from Sigma–Aldrich, Kanto Chemical, Tokyo Chemical Industry, and Wako Pure Chemical Industries, and used without further purification. Moisture-sensitive reactions were performed under an atmosphere of argon, unless otherwise noted. Reaction was monitored by thin-layer chromatography (TLC, Merck silica gel 60 F254 plate). Bands were visualized using UV light or by application of appropriate reagents (iodine vapor, phosphomolybdic acid-H_2SO_4, basic $KMnO_4$, and ninhydrin spray). Flash chromatography was carried out with silica gel (Silica gel 60N, 40–50 µm particle size) purchased from Kanto Chemical or Chromatorex NH silica gels purchased from Fuji Silysia Chemical. NMR spectra were recorded on a JEOL JNM-ECA500 (500 MHz) spectrometer, operating at 500 MHz for ^1H NMR and at 125MHz for ^{13}C NMR. Proton and carbon chemical shifts are expressed in δ values (ppm) relative to internal tetramethylsilane (0.00 ppm), residual $CHCl_3$ (7.26 ppm), CHD_2OD (3.31 ppm) or C_2HD_5SO (2.49 ppm) for ^1H NMR, and internal tetramethylsilane (0.00 ppm), $CDCl_3$ (77.16 ppm), methanol-*d*$_4$ (49.00 ppm) or dimethyl sulfoxide-*d*$_6$ (39.50 ppm) for ^{13}C NMR. Data are reported as follows: chemical shift, multiplicity (s, singlet; d, doublet; t, triplet; q, quartet; m, multiplet; br, broad), coupling constants (Hz), and integration. High-resolution mass spectrum was recorded using a Bruker micrOTOF II mass spectrometer.

Synthesis of itraAZY (Scheme S1-2)

2-chloro-4-iodoacetophenone (2). A suspension of 2-chloro-4-nitroacetophenone (0.50 g, 2.5 mmol) and NH_4Cl (1.35 g, 25.2 mmol) and iron powder (1.40 g, 25.1 mmol) in MeOH (35 mL) and H_2O (4 mL) was refluxed for 1 h. After the reaction, the reaction mixture was filtered through a Celite pad, and the filtrate was concentrated under reduced pressure to afford crude 2-chloro-4-aminoacetophenone (455 mg). The crude material was then added to 6 M H_2SO_4 (28 mL), and the mixture was stirred at 0 °C for 30 min. Sodium nitrite (557 mg, 8.07

mmol) was then added to the mixture at 0 °C, and the resulting mixture was further stirred at 0 °C for 10 min. To the mixture was added potassium iodide (1.34 g, 8.07 mmol) in cold H_2O (72 mL) at 0 °C, and the mixture was stirred at room temperature for 10 h. After the reaction, the mixture was extracted with AcOEt. The combined organic layer was washed with saturated aqueous sodium thiosulfate, dried over Na_2SO_4, and concentrated under reduced pressure. The residue was purified by silica gel column chromatography (hexane:DCM = 3:1 to 2:1) to afford the title compound (0.548 g, 1.96 mmol, 78% over 2 steps) as a white solid. ^{1}H-NMR (500 MHz, $CDCl_3$) δ 7.80 (d, *J* =1.7 Hz, 1H), 7.67 (dd, *J* = 8.0 1.7 Hz, 1H), 7.27 (d, *J* = 8.0 Hz, 1H), 2.63 (s, 3H); ^{13}C-NMR (125 MHz, $CDCl_3$) δ 199.39, 138.98, 138.24, 136.18, 132.19, 130.62, 97.61, 30.65; ESI-TOF-HRMS calcd for C_8H_6ClIO (*m/z*) $[M+Na]^+$ 302.9044, found 302.9044.

2-Bromo-2'-chloro-4'-iodoacetophenone (3). A solution of **2** (530 mg, 1.89 mmol), NBS (505 mg, 2.83 mmol), and PTSA (720 mg, 3.79 mmol) in MeCN (9.4 mL) was stirred at 60°C for 19 h. The reaction was partitioned between water and AcOEt, and the organic layer was washed with water and brine. The organic phase was dried over Na_2SO_4 and concentrated under reduced pressure. The residue (a yellow liquid, 731 mg), which contains the desired product as a major component along with di-brominated byproduct (mono:di = 6:1) and some succinimide, was used for the next reaction without further purification. ^{1}H-NMR (500 MHz, $CDCl_3$) δ 7.82 (d, *J* =1.7 Hz, 1H), 7.71 (dd, *J* = 8.0 1.5 Hz, 1H), 7.27 (d, *J* = 8.0 Hz, 1H), 4.47 (s, 2H).

4-Amino-1-(2-(2-chloro-4-iodophenyl)-2-oxoethyl)-1*H*-1,2,4-triazol-4-ium bromide (4). This reaction was performed by following the reported procedure (Astleford *et al.*, 1989) with slight modifications. The crude bromide **3** was dissolved in *i*PrOH (52 mL) and treated with 4-amino-1,2,4-triazole (480 mg, 5.71 mmol), and the reaction mixture was refluxed for 1.5 h. The resulting suspension was cooled to 0 °C and the pale yellow precipitate was collected by filtration to afford the title compound (520 mg, 1.18 mmol, 62% over 2 steps); ^{1}H-NMR (500 MHz, DMSO-*d$_6$*) δ 10.18 (s, 1H), 9.33 (s. 1H), 8.10 (d, *J* = 1.5 Hz, 1H), 7.99 (dd, *J* = 8.0, 1.5 Hz, 1H), 7.78 (d, *J* = 8.0 Hz, 1H), 7.16 (s, 2H), 6.17 (s, 2H); ^{13}C-NMR (125 MHz, DMSO-*d$_6$*) δ 190.71, 145.27, 144.12, 139.17, 136.55, 132.42, 132.20, 131.92, 102.01, 59.84; ESI-TOF-HRMS calcd for $C_{10}H_9ClIN_4O$ (*m/z*) $[M]^+$ 362.9504, found 362.9508.

1-(2-Chloro-4-iodophenyl)-2-(1*H*-1,2,4-triazol-1-yl)ethan-1-one (5). This reaction was performed by following the reported procedure (Astleford *et al.*, 1989) with slight modifications. A solution of **4** (511 mg, 1.16 mmol) in H_2O (3.0 mL) and concentrated HCl aq.

(5.2 mL) was treated with $NaNO_2$ (119 mg, 1.72 mmol) at 0 °C, and stirred for 5 min at the temperature. The reaction was allowed to warm up to the ambient temperature, and stirred for 1 h. The reaction was neutralized with saturated aqueous $NaHCO_3$, extracted with $CHCl_3$, dried over Na_2SO_4, and concentrated under reduced pressure. The residue was purified by silica gel column chromatography ($CHCl_3$/MeOH = 20:1) to afford the title compound (402 mg, 1.16 mmol, quant.) as a white solid. ^{1}H-NMR (500 MHz, DMSO-d_6) δ 8.53 (s, 1H), 8.05 (d, *J* = 1.7 Hz, 1H), 8.02 (s. 1H), 7.92 (dd, *J* = 8.0, 1.7 Hz, 1H), 7.67 (d, *J* = 8.0 Hz, 1H), 5.82 (s, 2H); ^{13}C-NMR (125 MHz, DMSO-d_6) δ 193.80, 151.53, 145.58, 138.71, 136.36, 134.12, 131.53, 131.17, 100.43, 57.06; ESI-TOF-HRMS calcd for $C_{10}H_7ClIN_3O$ (*m/z*) $[M-H]^-$ 345.9250, found 345.9237.

((2*SR*,4*RS*)-2-((1*H*-1,2,4-triazol-1-yl)methyl)-2-(2-chloro-4-iodophenyl)-1,3-dioxolan-4-yl)methanol (6). This compound was prepared by following a reported procedure (Tanoury *et al.*, 2003). Briefly, a solution of **5** (400 mg, 1.15 mmol) and 1-tosylglycerol (312 mg, 1.27 mmol) in anhydrous toluene (12 mL) was treated with TfOH (0.40 mL, 4.52 mmol) and stirred at the ambient temperature for 3 days. The reaction was quenched by adding aqueous 4 N NaOH, extracted with AcOEt, and concentrated under reduced pressure. The residue was treated with 1 M PTSA in AcOEt, and the resulted white precipitate was collected by filtration. Repeated recrystallization from MeCN gave a tosylate salt of **6** (383 mg, 0.513 mmol, 49%) as an essentially pure trans isomer as a white solid. To obtain free form of **6**, the tosylate salt was partitioned between AcOEt and saturated aqueous $NaHCO_3$, and the organic layer was washed with saturated aqueous $NaHCO_3$ and brine, dried over Na_2SO_4, and concentrated under reduced pressure to afford the title compound (290 mg, 0.504 mmol, 44%). Tosylate salt of **6**: ^{1}H-NMR (500 MHz, DMSO-d_6) δ 8.56 (s, 1H), 7.99 (s, 1H), 7.87 (d, *J* = 1.7 Hz, 1H), 7.80 (d, *J* = 8.5 Hz, 2H), 7.66 (dd, *J* = 8.0, 1.7 Hz, 1H), 7.51 (d, *J* = 8.5 Hz, 2H), 7.47 (d, *J* = 8.5 Hz, 2H), 7.12-7.10 (m, 3H), 4.74 (s, 2H), 4.23-4.19 (m, 1H), 3.93 (dd, *J* = 10.8, 4.0 Hz, 1H), 3.80-3.74 (m, 2H), 3.61 (dd, *J* = 8.6, 5.1 Hz, 1H), 2.43 (s, 3H), 2.28 (s, 3H); ^{13}C-NMR (125 MHz, DMSO-d_6) δ 149.6, 145.5, 145.2, 144.9 138.7, 137.7, 136.0, 134.9, 132.3, 132.0, 130.2, 130.1, 128.1, 127.7, 125.5, 107.9, 97.1, 74.1, 70.0, 66.2, 53.6, 21.6, 21.3; ESI-TOF-HRMS calcd for $C_{20}H_{19}ClIN_3O_5S$ (*m/z*) $[M+Na]^+$ 597.9671, found 597.9666. Free **6**: ^{1}H-NMR (500 MHz, $CDCl_3$) δ 8.06 (s, 1H), 7.75–7.74 (m, 4H), 7.53 (dd, *J* = 8.5, 1.5 Hz, 1H), 7.36 (d, *J* = 9.0 Hz, 2H), 7.17 (d, *J* = 8.0 Hz, 1H), 4.73 (d, *J* = 14.8 Hz, 1H), 4.65 (d, *J* = 14.8 Hz, 1H), 4.24–4.20 (m, 1H), 3.82–3.77 (m, 2H), 3.67 (dd, *J* = 8.5, 4.5 Hz, 1H), 3.50 (dd, *J* = 10.3, 6.3 Hz, 1H), 2.45 (s, 3H). An NOE correlation between the 7.17 peak and 4.25–4.20 peak was observed to confirm the indicated stereochemistry.

((2*SR*,4*RS*)-2-((1*H*-1,2,4-Triazol-1-yl)methyl)-2-(2-chloro-4-iodophenyl)-1,3-dioxolan-4-yl)methanol (7). To a solution of **6** (50.0 mg, 0.0870 mmol) in DMF (2.0 mL) was added sodium acetate (71.3 mg, 0.0869 mmol) at room temperature, and the mixture was refluxed for 17 h. After the reaction, the reaction mixture was extracted with hexane and AcOEt (4:1). The combined organic layer was washed with brine, dried over Na_2SO_4 and concentrated under reduced pressure to afford a crude material. The crude material was dissolved in MeOH (2.0 mL), treated with Cs_2CO_3 (141 mg, 0.433 mmol), and stirred for 1 h at the ambient temperature. After the reaction, the mixture was extracted with AcOEt. The combined organic layer was washed with brine, dried over Na_2SO_4 and concentrated under reduced pressure to afford the title product (36.5 mg, 0.0867 mmol, quant. over 2 steps) as a white solid. ^{1}H-NMR (500 MHz, $CDCl_3$) δ 8.12 (s, 1H), 7.96 (s, 1H), 7.81 (d, *J* = 1.7 Hz, 1H), 7.61 (dd, *J* = 8.6, 1.7 Hz, 1H), 7.34 (d, *J* = 8.6 Hz, 1H), 4.76 (s, 2H), 4.16-4.12 (m, 1H), 3.84 (dd, *J* = 8.0, 6.8 Hz, 1H), 3.70-3.65 (m, 2H), 3.25-3.23 (m, 1H); ^{13}C-NMR (125 MHz, $CDCl_3$) δ 151.84, 145.18, 139.85, 136.22, 135.42, 133.06, 129.95, 107.31, 95.49, 76.74, 66.14, 61.52, 53.27; ESI-TOF-HRMS calcd for $C_{13}H_{13}ClIN_3O_3$ (*m/z*) $[M+H]^+$ 421.9763 and $[M+Na]^+$ 443.9582, found 421.9748 and 443.9578.

((2*SR*,4*RS*)-2-((1*H*-1,2,4-Triazol-1-yl)methyl)-2-(4-azido-2-chlorophenyl)-1,3-dioxolan-4-yl)methanol (8). To a degassed solution of NaN_3 (23.1 mg, 0.355 mmol), trans-N,N'-dimethylcyclohexane-1,2-diamine (3.8 mg, 0.027 mmol), CuI (3.4 mg, 0.018 mmol) and L-sodium ascorbate (1.8 mg, 0.091 mmol) in EtOH (2.5 mL) and H_2O (1.0 mL) was added **7** (74.9 mg, 0.178 mmol) at room temperature under argon. The reaction mixture was refluxed for 3 h. After the reaction, the mixture was partitioned between saturated aqueous $NaHCO_3$ and AcOEt. The extracted organic layer was dried over Na_2SO_4 and concentrated under reduced pressure. The residue was purified by silica gel column chromatography ($CHCl_3$/MeOH = 30:1) to afford the title product (49.2 mg, 0.146 mmol, 82%) as a white solid. ^{1}H-NMR (500 MHz, $CDCl_3$) δ 8.13 (s, 1H), 7.91 (s, 1H), 7.58 (d, *J* = 8.0 Hz, 1H), 7.09 (d, *J* = 2.0 Hz, 1H), 6.90 (dd, *J* = 2.0, 8.0 Hz, 1H), 4.75 (m, 2H), 4.15-4.11 (m, 1H), 3.84 (dd, *J* = 7.4, 7.4 Hz, 1H), 3.69 (dd, *J* = 6.3, 6.3 Hz, 1H), 3.59 (dd, *J* = 12.0, 3.4 Hz, 1H), 3.27 (dd, *J* = 12.0, 4.5 Hz, 1H); ^{13}C-NMR (125 MHz, $CDCl_3$) δ 151.53, 145.04, 142.38, 133.35, 131.94, 129.81, 121.92, 117.37, 107.24, 76.72, 66.24, 61.53, 53.54; ESI-TOF-HRMS calcd for $C_{13}H_{13}ClN_6O_3$ (*m/z*) $[M+Na]^+$ 359.0630, found 359.0631.

((2*SR*,4*SR*)-2-((1*H*-1,2,4-Triazol-1-yl)methyl)-2-(4-azido-2-chlorophenyl)-1,3-dioxolan-4-yl)methyl 4-methylbenzenesulfonate (9). To a solution of **8** (43 mg, 0.13 mmol) and TsCl (68 mg, 0.36 mmol) in DCE (2.3 mL) was slowly added Et_3N (0.050 mL, 0.36 mmol) at 0 °C,

and the mixture was stirred for 16 h at 60 °C. After the reaction, the mixture was quenched with 1 N HCl, and the resulting mixture was extracted with AcOEt. The combined organic layer was washed with 5 N NaOH and 1 N HCl, dried over Na_2SO_4, and concentrated under reduced pressure. The residue was purified by silica gel column chromatography ($CHCl_3$/MeOH = 40:1) to afford the title product (63 mg, 0.13 mmol, quant.) as a white solid. ^{1}H-NMR (500 MHz, $CDCl_3$) δ 8.06 (s, 1H), 7.76-7.75 (m, 3H), 7.43 (d, J = 8.6 Hz, 1H), 7.36 (d, J = 8.0 Hz, 2H), 7.05 (d, J = 2.2 Hz, 1H), 6.85 (dd, J = 8.6, 2.2 Hz, 1H), 4.74 (d, J = 14.5 Hz, 1H), 4.67 (d, J = 14.5 Hz, 1H), 4.26-4.20 (m, 1H), 3.84-3.78 (m, 2H), 3.68 (dd, J = 8.6, 4.6 Hz, 1H), 3.52 (dd, J = 10.3, 6.3 Hz, 1H), 2.45 (s, 3H); ESI-TOF-HRMS calcd for $C_{20}H_{19}ClN_6O_5S$ (*m/z*) [M+Na]$^+$ 513.0718, found 513.0718.

4-(4-(4-(4-Methoxyphenyl)piperazin-1-yl)phenyl)-2,4-dihydro-3*H*-1,2,4-triazol-3-one (13). This compound, along with the intermediates **11** and **12**, were prepared as described previously (Heeres *et al.*, 1984; Shi *et al.*, 2010; Pace *et al.*, 2016). A purple solid (76% over 3 steps). ^{1}H-NMR (500 MHz, DMSO-d_6) δ 8.24 (s, 1H), 7.47 (d, J = 9.0 Hz, 2H), 7.09 (d, J = 9.0 Hz, 2H), 6.96 (d, J = 9.0 Hz, 2H), 6.84 (d, J = 9.0 Hz, 2H), 3.69 (s, 3H), 3.32-3.29 (m, 4H), 3.16-3.14 (m, 4H); ESI-TOF-HRMS calcd for $C_{19}H_{21}N_5$ (*m/z*) [M+Na]$^+$ 374.1587, found 374.1571.

4-(4-(4-(4-(((2*RS*,4*SR*)-2-((1*H*-1,2,4-Triazol-1-yl)methyl)-2-(2,4-dichlorophenyl)-1,3-dioxolan-4-yl)phenyl)-2,4-dihydro-3*H*-1,2,4-triazol-3-one (14). A solution of **13** (300 mg, 0.854 mmol), 4-bromo-1-butyne (0.230 mL, 2.58 mmol), and Cs_2CO_3 (1.38 g, 4.24 mmol) in DMF (4.2 mL) was stirred and heated to 100°C for 24 h. The reaction mixture was diluted with H_2O and the resulting pale purple precipitate was collected by filtration, and washed with H_2O. The precipitate was extracted with $CHCl_3$ and purified by silica gel column chromatography ($CHCl_3$/AcOEt/MeOH = 45:10:1) to afford the title compound (230 mg, 0.570 mmol, 67%) as a pale brown solid. It was noted that two peaks could not be observed under normal ^{13}C-NMR condition but could be clearly observed with HMBC measurement. ^{1}H-NMR (500 MHz, $CDCl_3$) δ 7.62 (s, 1H), 7.40 (d, J = 8.9 Hz, 2H), 7.03 (d, J = 8.9 Hz, 2H), 6.97 (d, J = 8.3 Hz, 2H), 6.87 (d, J = 8.3 Hz, 2H), 4.04 (t, J = 7.2 Hz, 2H), 3.78 (s, 3H), 3.39–3.43 (m, 4H), 3.25–3.22 (m, 4H), 2.70 (td, J = 7.2 Hz, 2.9 Hz, 2H), 2.03 (t, J = 2.9 Hz, 1H); ^{13}C-NMR (125 MHz, $CDCl_3$) δ 154.12 (from HMBC), 152.01, 150.66, 145.22 (from HMBC), 134.43, 125.56, 123.60 (2C), 118.63 (2C), 116.60 (2C), 114.50 (2C), 80.29, 70.22, 55.54, 50.80 (br, 2C), 49.13 (2C), 44.19, 18.66. ESI-TOF-HRMS calcd for $C_{23}H_{25}N_5O_2$ (*m/z*) [M+H]$^+$ 404.2081 and [M+Na]$^+$ 426.1900, found 404.2063 and 426.1891; IR (KBr) 3271, 3056, 2833, 1688, 1560, 1514, 1228, 1030, 818 cm^{-1}.

2-(But-3-yn-1-yl)-4-(4-(4-(4-hydroxyphenyl)piperazin-1-yl)phenyl)-2,4-dihydro-3*H*-1,2,4-triazol-3-one (15). A solution of the methyl ether **14** (230 mg, 0.570 mmol) in DCM (11.4 mL) was treated with BBr_3 (1 M in DCM, 6.8 mL, 6.8 mmol) at 0ºC, and stirred for 16 h at the ambient temperature. The reaction was quenched by adding MeOH followed by saturated aqueous $NaHCO_3$, and extracted with DCM. The extract was concentrated and purified by silica gel column chromatography ($CHCl_3$/MeOH = 30:1) to afford the title compound (147 mg, 0.378 mmol, 66%) as a white solid. ^{1}H-NMR (500 MHz, $CDCl_3$) δ ^{1}H-NMR ($CDCl_3$–CD_3OD, 10:1) δ: 7.61 (s, 1H), 7.31 (d, *J* = 8.0 Hz, 2H), 6.97 (d, *J* = 8.0 Hz, 2H), 6.84 (d, *J* = 8.0 Hz, 2H), 6.72 (d, *J* = 8.0 Hz, 2H), 3.96 (t, *J* = 6.9 Hz, 2H), 3.31–3.27 (m, 4H), 3,15–3.10 (m, 4H), 2.63 (td, *J* = 6.9, 2.8 Hz, 2H), 1.99 (t, *J* = 2.8 Hz, 1H); ^{13}C-NMR (125 MHz, $CDCl_3$–CD_3OD, 10:1) δ 152.15, 151.31, 150.70, 144.34, 134.76, 125.07, 123.74 (2C), 119.01 (2C), 116.43 (2C), 115.64 (2C), 79.93, 70.23, 51.05 (2C), 48.91 (2C), 44.11, 18.43.

4-(4-(4-(4-(((2*R*,4*S*)-2-((1*H*-1,2,4-triazol-1-yl)methyl)-2-(4-azido-2-chlorophenyl)-1,3-dioxolan-4-yl)methoxy)phenyl)piperazin-1-yl)phenyl)-2-(but-3-yn-1-yl)-2,4-dihydro-3*H*-1,2,4-triazol-3-one (itraAZY). A suspension of **15** (22.6 mg, 0.0581 mmol), **8** (30.0 mg, 0.0612 mmol), and Cs_2CO_3 (37.9 mg, 0.116 mmol) in DMSO was heated to 90ºC and stirred for 1.5 h. The reaction mixture was diluted with AcOEt, washed with brine, dried over Na_2SO_4, and concentrated under reduced pressure. Silica gel column chromatography (Chromatorex NH silica gels, $CHCl_3$/hexane/AcOEt = 5:2:1) followed by crystallization from MeOH with small amount of DCM gave the title compound (10.3 mg, 0.0146 mmol, 25%) as a white solid. ^{1}H-NMR (500 MHz, $CDCl_3$) δ 8.20 (s, 1H), 7.89 (s, 1H), 7.62 (s, 1H), 7.60 (d, *J* = 8.0 Hz, 1H), 7.40 (d, *J* = 9.0 Hz, 2H), 7.11 (d, *J* = 2.2 Hz, 1H), 7.03 (d, *J* = 9.0 Hz, 2H), 6.94 (d, *J* = 9.0 Hz, 2H), 6.92 (dd, *J* = 8.0, 2.2 Hz, 1H), 6.80 (d, *J* = 9.0 Hz, 2H), 4.84 (d, *J* = 14.8 Hz, 1H), 4.76 (d, *J* = 14.9 Hz, 1H), 4.38-4.34 (m, 1H), 4.03 (t, *J* = 7.1 Hz, 2H), 3.92 (dd, *J* = 8.0, 6.3 Hz, 2H), 3.83-3.79 (m, 2H), 3.49 (dd, *J* = 9.7, 6.3 Hz, 2H), 3.37-3.35 (m, 4H), 3.24-3.22 (m, 4H), 2.70 (dt, *J* = 2.8, 7.4 Hz, 2H), 2.02 (t, *J* = 2.5 Hz, 2H); ^{13}C-NMR (125 MHz, $CDCl_3$) δ152.62, 152.04, 151.37, 150.70, 145.99, 144.88, 142.60, 140.91, 134.45, 131.84, 129.88, 125.60, 123.64, 122.00, 118.47, 117.42, 116.64, 115.27, 107.69, 80.30, 74.65, 70.21, 67.70, 67.42, 53.74, 50.58, 49.16, 44.22, 18.69 ; ESI-TOF-HRMS calcd for $C_{35}H_{34}Cl_2N_8O_4$ (*m/z*) $[M+Na]^+$ 730.2376; found, 730.2352.

Synthesis of itraACT (Scheme S3)

1-(2,4-Dichlorophenyl)-2-(1*H*-1,2,4-triazol-1-yl)ethan-1-one (17). This compound was prepared in a similar manner to the compound **5**. A white solid (63% over 2 steps); ^{1}H-NMR

(500 MHz, $CDCl_3$) δ 8.23 (s, 1H), 7.98 (s, 1H), 7.65 (d, *J* = 8.0 Hz, 1H), 7.50 (d, *J* = 1.7 Hz, 1H), 7.38 (dd, *J* = 8.0, 1.7 Hz, 1H), 5.62 (s, 2H).

((2*SR*,4*SR*)-2-((1*H*-1,2,4-Triazol-1-yl)methyl)-2-(2,4-dichlorophenyl)-1,3-dioxolan-4-yl)methyl 4-methylbenzenesulfonate (18-trans). This compound was prepared in a similar manner to the compound **6**, by following a reported procedure. The desired isomer **18-trans** was obtained as a white solid (22%) along with the other isomer, **18-cis** (5.4%). **18-trans tosylate salt**: ^{1}H-NMR (500 MHz, DMSO-d_6) δ 8.39 (s, 1H), 7.86 (s, 1H), 7.81 (d, *J* = 8.5 Hz, 2H), 7.65 (d, *J* = 1.7 Hz, 1H), 7.51 (d, *J* = 8.0 Hz, 2H), 7.47 (d, *J* = 7.5 Hz, 2H), 7.38 (dd, *J* = 8.5, 1.7 Hz, 1H), 7.34 (d, *J* = 8.5 Hz, 1H), 7.11 (d, *J* = 7.5 Hz, 2H), 4.74 (s, 2H), 4.25-4.20 (m, 1H), 3.94 (dd, *J* = 10.9, 4.0 Hz, 1H), 3.82-3.76 (m, 2H), 3.63 (d, *J* = 8.6, 5.1 Hz, 1H), 2.43 (s, 3H), 2.29 (s, 3H). **18-trans free form**: ^{1}H-NMR (500 MHz, $CDCl_3$) δ 8.08 (s, 1H), 7.79 (d, *J* = 8.5 Hz, 2H), 7.78 (s, 1H), 7.45 (d, *J* = 1.7 Hz, 1H), 7.43 (d, *J* = 8.0 Hz, 1H), 7.39 (d, *J* = 8.0 Hz, 2H), 7.21 (d, *J* = 7.5 Hz, 1H), 4.76 (d, *J* = 15.0 Hz, 1H), 4.69 (d, *J* = 15.0 Hz, 1H), 4.28-4.23 (m, 1H), 3.86-3.80 (m, 2H), 3.71 (dd, *J* = 8.5, 4.5 Hz, 1H), 3.53 (dd, *J* = 9.5, 5.0 Hz, 1H), 2.47 (s, 3H); ESI-TOF-HRMS calcd for $C_{20}H_{19}Cl_2N_3O_5S$ (*m/z*) $[M+H]^+$ 484.0495 and $[M+Na]^+$ 506.0315, found 484.0506 and 506.0326.

Ethyl 2-(4-(4-(4-methoxyphenyl)piperazin-1-yl)phenyl)-2*H*-tetrazole-5-carboxylate (21). This compound was prepared by following the reported procedure (Herner *et al.*, 2016). A solution of 47% w/w ethyl 2-oxoacetate in toluene (0.75 mL, 3.56 mmol) and benzenesulfonyl hydrazide (455 mg, 2.64 mmol) in EtOH (19 mL) was stirred at r.t. for 1 h. The solvent was removed under reduced pressure and the crude **20** was re-dissolved in pyridine (19 mL). Separately, a suspension of 4-(4-(4-methoxyphenyl)piperazin-1-yl)aniline (500 mg, 1.77 mmol) and concentrated HCl (0.66 mL) in H_2O (1.7 mL) and EtOH (1.3 mL) was treated dropwise with a solution of sodium nitrite (134 mg, 1.93 mmol) in H_2O (0.5 mL) at 0 °C, and the reaction mixture was stirred for 10 min. This solution was added dropwise to the pyridine solution of **20** over 50 min at –10 °C, and the mixture was warmed up to room temperature over 2 h. To the mixture was then added cold water, and the resulting mixture was further stirred at 0 °C for 30 min. The brown solid was collected by filtration, washed with water, and dissolved in AcOEt. The solution was concentrated under reduced pressure, and purified by silica gel column chromatography (hexane/AcOEt = 3:1) to afford the title product (206 mg, 0.505 mmol, 29%) as a brown solid. ^{1}H-NMR (500 MHz, $CDCl_3$) δ 8.07 (d, *J* = 9.1 Hz, 2H), 7.06 (d, *J* = 9.1 Hz, 2H), 6.96 (d, *J* = 8.5 Hz, 2H), 6.87 (d, *J* = 8.5 Hz, 2H), 4.57 (q, *J* = 7.2 Hz, 2H), 3.79 (s, 3H), 3.48-3.46 (m, 4H), 3.25-3.23 (m, 4H), 1.49 (t, *J* = 7.2 Hz, 3H); ^{13}C-NMR (125 MHz, $CDCl_3$) δ 158.01, 157.36, 154.29, 152.44, 145.29, 128.14, 121.47 (2C), 118.67

(2C), 115.45 (2C), 114.53 (2C), 62.67, 55.57, 50.70 (2C), 48.35 (2C), 14.22; ESI-TOF-HRMS calcd for $C_{21}H_{24}N_6O_3$ (*m/z*) $[M+H]^+$ 409.1983 and $[M+Na]^+$ 431.1802, found 409.1950 and 431.1773.

2-(4-(4-(4-Methoxyphenyl)piperazin-1-yl)phenyl)-*N*-(prop-2-yn-1-yl)-2*H*-tetrazole-5-carboxylic acid (22). A solution of **21** (50 mg, 0.122 mmol) in THF (1 mL) and EtOH (1 mL) was treated with 5 N NaOH (0.24 mL, 1.2 mmol) at room temperature, and the mixture was stirred for 16 h. The mixture was then neutralized by an addition of cold 2 N HCl aq. and cold water, and the resulting mixture was further stirred at 0 °C for 30 min. The yellow solid was collected by filtration, washed with water and dissolve in MeOH. The residue was concentrated under reduced pressure to afford the crude **22** (43.1 mg). This material was used for the next reaction without further purification. ^{1}H-NMR (500 MHz, DMSO-d_6) δ 7.91 (d, J = 9.5 Hz, 2H), 7.23 (d, J = 9.5 Hz, 2H), 6.97 (d, J = 9.1 Hz, 2H), 6.85 (d, J = 9.1 Hz, 2H), 3.70 (s, 3H), 3.46-3.44 (m, 4H), 3.18-3.16 (m, 4H); ESI-TOF-HRMS calcd for $C_{19}H_{20}N_6O_3$ (*m/z*) $[M+Na]^+$ 381.1670, found 381.1663.

2-(4-(4-(4-Methoxyphenyl)piperazin-1-yl)phenyl)-*N*-(prop-2-yn-1-yl)-2*H*-tetrazole-5-carboxamide (23). The crude acid **22** (40.0 mg, 0.105 mmol) was dissolved in DMF (2.1 mL) and treated with HATU (80 mg, 0.210 mmol) and DIPEA (0.056 mL, 0.031 mmol), and stirred at room temperature for 30 min. Propargylamine (0.013 mL, 0.21 mmol) was then added to the mixture, and the reaction was stirred at room temperature for 20 min. The resulting mixture was further stirred at 90 °C for 2 h. After the reaction, the mixture was diluted with cold water, and the resulting mixture was further stirred at 0 °C for 30 min. The yellow solid was collected by filtration, washed with water and dissolved in $CHCl_3$. The residue was concentrated under reduced pressure, and purified by silica gel column chromatography ($CHCl_3$/AcOEt = 4:1) to afford the title compound (30.7 mg, 0.0736 mmol, 60% over 2 steps) as a yellow solid. ^{1}H-NMR (500 MHz, $CDCl_3$) δ 8.06 (d, J = 9.2 Hz, 2H), 7.38 (t, J = 5.1 Hz, 1H), 7.05 (d, J = 9.2 Hz, 2H), 6.96 (d, J = 9.1 Hz, 2H), 6.87 (d, J = 9.1 Hz, 2H), 4.35 (dd, J = 5.1, 2.3 Hz, 2H), 3.78 (s, 3H) 3.47-3.46 (m, 4H), 3.25-3.23 (m, 4H), 2.32 (t, J = 2.3 Hz, 1H); ^{13}C-NMR (125 MHz, $CDCl_3$) δ 158.82, 156.33, 154.27, 152.40, 145.28, 128.13, 121.38 (2C), 118.65 (2C), 115.44 (2C), 114.52 (2C), 78.37, 72.46, 55.56, 50.68 (2C), 48.34 (2C), 29.33; ESI-TOF-HRMS calcd for $C_{22}H_{23}N_7O_2$ (*m/z*) $[M+Na]^+$ 440.1805, found 440.1798.

2-(4-(4-(4-Hydroxyphenyl)piperazin-1-yl)phenyl)-*N*-(prop-2-yn-1-yl)-2*H*-tetrazole-5-carboxamide (24). A solution of **23** (30.0 mg, 0.0719 mmol) in DCM (3.6 mL) was treated with BBr_3 (1 M in DCM, 0.70 mL, 0.70 mmol) at 0 °C, and the reaction was stirred at the

ambient temperature for 22 h. The reaction was quenched with saturated aqueous $NaHCO_3$ and MeOH, and extracted with DCM. The extract was concentrated under reduced pressure, and the residue was purified by silica gel column chromatography ($CHCl_3$/AcOEt = 10:1) to afford the title compound (27.4 mg, 0.0680 mmol, 95%) as a pale yellow solid. ^{1}H-NMR (500 MHz, acetone-d_6) δ 8.60 (t, J = 5.0 Hz, 1H), 8.00 (d, J = 9.1 Hz, 2H), 7.86 (br s, 1H), 7.25 (d, J = 9.1 Hz, 2H), 6.92 (d, J = 8.6 Hz, 2H), 6.77 (d, J = 8.6 Hz, 2H), 4.28 (dd, J = 5.0, 2.3 Hz, 2H), 3.51-3.49 (m, 4H), 3.21-3.19 (m, 4H), 2.73 (t, J = 2.3Hz, 1H); ^{13}C-NMR (125 MHz, acetone-d_6) δ 160.38, 157.08, 153.59, 152.54, 145.81, 128.83, 122.13 (2C), 119.57 (2C), 116.47 (2C), 116.28 (2C), 80.74, 72.23, 51.58 (2C), 49.01 (2C), 29.16; ESI-TOF-HRMS calcd for $C_{21}H_{21}N_7O_2$ (*m/z*) $[M+Na]^+$ 426.1649, found 426.1640.

2-(4-(4-(4-(((2*RS*,4*SR*)-2-((1*H*-1,2,4-Triazol-1-yl)methyl)-2-(2,4-dichlorophenyl)-1,3-dioxolan-4-yl)methoxy)phenyl)piperazin-1-yl)phenyl)-*N*-(prop-2-yn-1-yl)-2*H*-tetrazole-5-carboxamide (itraACT). A suspension of **24** (13.0 mg, 0.0322 mmol), **18-trans** (18.0 mg, 0.0373 mmol) and Cs_2CO_3 (20 mg, 0.061 mmol) in DMF (1.6 mL) was heated to 90 °C for 2 h, and the reaction mixture was diluted with H_2O. The mixture was extracted with AcOEt, the organic layer was washed with brine, dried over Na_2SO_4, and concentrated under reduced pressure. The residue was purified by silica gel column chromatography (Chromatorex NH silica gels, $CHCl_3$/hexane/AcOEt = 5:2:1) to afford the title compound (13.1 mg, 0.0183 mmol, 57%) a white solid. ^{1}H-NMR (500 MHz, $CDCl_3$) δ 8.20 (s, 1H), 8.07 (d, J = 9.2 Hz, 2H), 7.89 (s, 1H), 7.57 (d, J = 8.5 Hz, 1H), 7.47 (d, J = 2.5 Hz, 1H), 7.38 (t, J = 5.4 Hz, 1H), 7.25 (dd, J = 8.5, 2.5 Hz, 1H), 7.06 (d, J = 9.2 Hz, 2H), 6.94 (d, J = 9.1 Hz, 2H), 6.80 (d, J = 9.1 Hz, 2H), 4.84 (d, J = 14.8 Hz, 1H), 4.76 (d, J = 14.8 Hz, 1H), 4.39-4.34 (m, 3H), 3.91 (dd, J = 8.6, 6.9 Hz, 1H), 3.83-3.78 (m, 2H), 3.50-3.46 (m, 4H), 3.25-3.3.23 (m, 4H), 2.32 (t, J = 2.2 Hz, 1H); ^{13}C-NMR (125 MHz, $CDCl_3$) δ 158.84, 156.33, 152.72, 152.39, 151.38, 145.84, 144.91, 136.07, 134.03, 133.11, 131.44, 129.59, 128.17, 127.25, 121.40 (2C), 118.52 (2C), 115.48 (2C), 115.28 (2C), 107.62, 78.37, 74.68, 72.48, 67.63, 67.42, 53.57, 50.48 (2C), 48.31 (2C), 29.34; ESI-TOF-HRMS calcd for $C_{34}H_{32}Cl_2N_{10}O_4$ (*m/z*) $[M+H]^+$ 715.2058 and $[M+Na]^+$ 737.1877, found 715.2021 and 737.1848.

Synthesis of itraBIO (Scheme S4)

D-Biotin pentafluorophenyl ester (biotin-PFP). This compound was prepared as previously reported (Jones *et al.*, 2012). Briefly, a solution of D-biotin (405 mg, 1.66 mmol) and Et_3N (0.345 mL, 2.49 mmol) in DMF (10 mL) was treated with pentafluorophenyl trifluoroacetate (0.425 mL, 2.49 mmol), and the mixture was stirred for 3 h at room temperature. The reaction mixture was diluted with diethyl ether (20 mL) and the white

precipitates were collected by filtration to afford the title product (682 mg, 1.66 mmol, quant.) as a colorless powder.

***N*-(35-azido-3,6,9,12,15,18,21,24,27,30,33-undecaoxapentatriacontyl)-5-((3a*R*,4*R*,6a*S*)-2-oxohexahydro-1*H*-thieno[3,4-*d*]imidazol-4-yl)pentanamide (25).** To a solution of **biotin-PFP** (43.0 mg, 0.105 mmol) and N_3-PEG_{11}-amine (50.0 mg, 0.0876 mmol) in DMF (1.7 mL) was added Et_3N (0.024 mL, 17 mmol), and the mixture was stirred for 24 h at room temperature After the reaction, the mixture was concentrated under reduced pressure, purified by silica gel column chromatography ($CHCl_3$/MeOH = 25:1 to 0:1) to afford the title compound (68.8 mg, 0.0864 mmol 82%) as a colorless liquid. ESI-TOF-HRMS calcd for $C_{34}H_{64}N_6O_{13}S$ (*m/z*) $[M+Na]^+$ 819.4144; found, 819.4119.

4-(4-(4-(4-(((2*RS*,4*S*)R-2-((1*H*-1,2,4-Triazol-1-yl)methyl)-2-(2,4-dichlorophenyl)-1,3-dioxolan-4-yl)methoxy)phenyl)piperazin-1-yl)phenyl)-2-(but-3-yn-1-yl)-2,4-dihydro-3*H*-1,2,4-triazol-3-one (26). A solution of **15** (13.5 mg, 0.0347 mmol) and **18-trans** (19.8 mg, 0.0469 mmol) in DMF (0.5 mL) was treated with Cs_2CO_3 (21.6 mg, 0.0663 mmol) under Ar and heated to 100°C for 3 h. The reaction was diluted with MeOH (1 mL) and H_2O (3 mL) and the precipitate was collected by filtration, followed by brief washes with MeOH-H_2O (1:1). The solid was purified by silica gel column chromatography (Chromatorex NH silica gels, hexane/DCM/AcOEt = 1:2:2) to afford the title compound (16.5 mg, 0.0235 mmol, 68%) as a white solid.^{1}H-NMR (500 MHz, $CDCl_3$) δ 8.21 (s, 1H), 7.90 (s, 1H), 7.63 (s, 1H), 7.57 (d, *J* = 8.6 Hz, 1H), 7.47 (d, *J* = 2.3 Hz, 1H), 7.40 (d, *J* = 9.2 Hz, 2H), 7.03 (d, *J* = 8.6 Hz, 2H), 6.94 (d, *J* = 8.6 Hz, 2H), 6.80 (d, *J* = 9.2 Hz, 2H), 4.84 (d, *J* = 14.9 Hz, 1H), 4.76 (d, *J* = 14.9 Hz, 1H), 4.39–4.34 (m, 1H), 4.04 (t, *J* = 7.2 Hz, 2H), 3.92 (t, *J* = 7.4 Hz, 1H), 3.83-3.79 (m, 2H), 3.49 (dd, *J* = 9,7, 6.3 Hz, 1H), 3.38–3.35 (m, 4H), 3.25–3.22 (m, 4H), 2.70 (td, *J* = 7.2, 6.3 Hz, 2H), 2.03 (t, *J* = 2.6 Hz, 1H); ^{13}C-NMR (125 MHz, $CDCl_3$) δ 152.60, 152.02, 151.38, 150.67, 145.97, 144.91, 136.04, 134.43, 134.03, 133.10, 131.42, 129.59, 127.23, 125.58, 123.61 (2C), 118.45 (2C), 116.62 (2C), 115.25 (2C), 107.61, 80.30, 74.67, 70.22, 67.62, 67.42, 53.57, 50.55 (2C), 49.13 (2C), 44.20, 18.66.; ESI-TOF-HRMS calcd for $C_{35}H_{34}Cl_2N_8O_4$ (*m/z*) $[M+Na]^+$ 723.1972; found, 723.2007.

itraBIO (RS397). To a degassed solution of **26** (15.0 mg, 0.0214 mmol), **25** (25.0 mg, 0.0314 mmol) and CuI (2.0 mg, 0.010 mmol) in MeCN (2 mL) was added DIPEA (7.6 μL, 0.043 mmol) under argon. The reaction mixture was stirred at the ambient temperature for 5 days. The reaction was quenched with saturated aqueous NH_4Cl and extracted with AcOEt. The combined organic layer was dried over Na_2SO_4 and concentrated under reduced pressure.

The residue was purified by silica gel column chromatography ($CHCl_3$/MeOH = 15:1) to afford the title compound (15.7 mg, 0.0105 mmol, 49%) as a white solid. ^{1}H-NMR (500 MHz, $CDCl_3$) δ 8.21 (s, 1H), 7.89 (s, 1H), 7.64 (s, 1H), 7.61 (s, 1H), 7.57 (d, *J* = 8.0 Hz, 1H), 7.47 (d, *J* = 1.5 Hz, 1H), 7.39 (d, *J* = 8.5 Hz, 2H), 7.25 (dd, *J* = 8.0, 1.5 Hz, 1H), 7.02 (d, *J* = 8.0 Hz, 2H), 6.93 (d, *J* = 8.5 Hz, 2H), 6.80 (d, *J* = 8.5 Hz, 2H), 6.68 (br, 1H), 6.06 (br, 1H), 5.27 (br, 1H), 4.84 (d, *J* = 14.8 Hz, 1H), 4.76 (d, *J* = 14.8 Hz, 1H), 4.54–4.49 (m, 3H), 4.38–4.34 (m, 1H), 4.33–4.31 (m, 1H), 4.20–4.17 (m, 2H), 3.93–3.90 (m, 2H), 3.85–3.78 (m, 3H), 3.68–3.61 (m, 40H), 3.59–3.57 (m, 4H), 3.49 (dd, *J* = 9.5, 6.5 Hz, 1H), 3.42 (t, *J* = 5.0 Hz, 2H), 3.39–3.33 (m, 4H), 3.26–3.21 (m, 5H), 3.18–3.12 (m, 2H), 2.90 (dd, *J* = 13.0, 5.0 Hz, 1H), 2.73 (d, *J* = 13.0 Hz, 1H), 2.22 (t, *J* = 7.5 Hz, 2H), 1.78–1.59 (m, 4H), 1.47–1.41 (m, 2H); ^{13}C-NMR(125 MHz, $CDCl_3$) δ 173.14, 163.50, 152.59, 152.05, 151.37, 150.60, 148.20, 145.96, 144.10, 136.03, 134.22, 134.03, 133.10, 131.41, 129.59, 127.23, 125.70, 123.60, 122.57, 118.44, 116.61, 115.26, 107.60, 74.67, 70.71, 70.60, 70.51 (m), 70.41, 70.38, 70.08, 69.88, 69.55, 69.28, 67.62, 67.41, 61.73, 60.12, 55.39, 53.59, 50.56, 50.17, 49.15, 44.85, 44.66, 40.51, 39.11, 35.81, 29.66, 28.09, 25.47; ESI-TOF-HRMS calcd for $C_{69}H_{98}Cl_2N_{14}O_{17}S$ (*m/z*) $[M+Na]^+$ 1519.6224, found 1519.6267.

Synthesis of lapaAZY (Scheme S5)

(3-azidophenyl)methanol (28). To a solution of 3-aminobenzyl alcohol **27** (200 mg, 1.62 mmol) in 6 M HCl (2 mL) was added sodium nitrite (168 mg, 2.44 mmol) at 0 °C, and stirred for 5 min. Sodium azide (422mg, 6.49 mmol) was then added to the mixture, and stirred at 0 °C for 30 min, and the reaction mixture was extracted with AcOEt. The combined organic layer was washed with $NaHCO_3$ and brine, dried over Na_2SO_4, and concentrated under reduced pressure. The residue was purified by silica gel column chromatography (hexane/AcOEt = 5:1) to afford the title compound (242 mg, 1.62 mmol, quant.) as a pale yellow liquid. ^{1}H-NMR (500 MHz, $CDCl_3$) δ 7.34 (dd *J* = 7.5, 7.5 Hz, 1H), 7.13 (d, *J* = 7.5 Hz, 1H), 7.06 (br, 1H), 6.96 (dd, *J* = 7.5, 1.5 Hz, 1H), 4.70 (s, 2H), 1.76 (br, 1H); ^{13}C-NMR (125 MHz, $CDCl_3$) δ 142.87, 140.34, 129.92, 123.21, 118.20, 117.28, 64.74; IR (KBr, cm^{-1}) 3427, 2111 (strong), 1609, 1588, 1485, 1447, 1291.

1-azido-3-(chloromethyl)benzene (29). To a solution of **28** (237 mg, 1.59 mmol) and TsCl (909 mg, 4.77 mmol) in DCM (8 mL) was added pyridine (0.49 mL, 4.76 mmol) at 0 °C, and stirred for 5 min. The mixture was warmed to room temperature and further stirred for 15 h. After the reaction, the reaction mixture was quenched with 5 N NaOH, and extracted with AcOEt. The combined organic layer was washed with 2 N HCl, dried over Na_2SO_4 and concentrated under reduced pressure. The residue was purified by silica gel column

chromatography (hexane/AcOEt = 20:1) to afford the title compound (106 mg, 0.635 mmol, 40%) as a pale yellow liquid. ^{1}H-NMR (500 MHz, $CDCl_3$) δ 7.35 (dd, *J* = 7.8 Hz, 1H), 7.16 (d, *J* = 8.5 Hz, 1H), 7.06 (dd, *J* = 2.0, 2.0 Hz, 1H), 6.99 (dd, *J* = 8.0, 2.0 Hz, 1H), 4.56 (s, 2H); ^{13}C-NMR (125 MHz, $CDCl_3$) δ 140.53, 139.35, 130.13, 125.00, 119.08, 118.99, 45.49; IR (KBr, cm^{-1}) 2116 (strong), 1609, 1590, 1487, 1448, 1299.

5-(4-((4-((3-azidobenzyl)oxy)-3-chlorophenyl)amino)quinazolin-6-yl)furan-2-carbaldehyde (31). A solution of 5-[4-[3-Chloro-4-(3-fluorobenzyloxy)anilino]-6-quinazolinyl]furan-2-carboxaldehyde **30** (200 mg, 0.422 mmol) in DCM (8.4 mL) was treated with BBr_3 (1 M in DCM, 4.2 mL, 4.2 mmol) at 0 °C, and stirred for 5 min at the temperature. The reaction was allowed to warm up to ambient temperature and was stirred for 1.5 h. The reaction was quenched with saturated aqueous $NaHCO_3$ and MeOH, and extracted with AcOEt. The residue was purified by flash column silica gel chromatography ($CHCl_3$/acetone = 1:1) to afford the crude intermediate phenol (42.0 mg) as a pale yellow solid. This compound was almost insoluble in $CHCl_3$ or DMSO etc. and could not be characterized by ^{1}H or ^{13}C NMR. To a suspension of the crude phenol (40.0 mg, 0.109 mmol) and **29** (22.0 mg, 0.132 mmol) in DMF (2 mL) was added K_2CO_3 (45 mg, 0.33 mmol) at room temperature, and the mixture was stirred for 1 h at 80 °C.The mixture was treated with H_2O at 0 °C, and the resulting mixture was further stirred at 0 °C for 30 min. The yellow precipitates was collected by filtration, washed with water and dissolved with $CHCl_3$. The residue was purified by silica gel column chromatography ($CHCl_3$/MeOH = 30:1) to afford the title compound (40.0 mg, 0.0806 mmol, 19% over 2 steps) as a yellow solid. ^{1}H-NMR (500 MHz, $CDCl_3$) δ 9.68 (s, 1H), 8.74 (s, 1H), 8.46 (s, 1H), 8.08 (dd, *J* = 8.6, 1.1 Hz, 1H), 7.94 (d, *J*= 9.0 Hz, 1H), 7.92 (d, *J* = 2.5 Hz, 1H), 7.85 (br s, 1H), 7.58 (dd, *J* = 8.6, 2.3 Hz, 1H), 7.40-7.37 (m, 2H), 7.25 (d, *J* = 8.0 Hz, 1H), 7.18 (s, 1H), 7.01-6.99 (m, 3H), 5.16 (s, 2H); ^{13}C-NMR (125 MHz, $CDCl_3$) δ 177.15, 160.95, 158.04, 157.82, 155.81, 152.26, 151.39, 150.65, 140.51, 138.56, 131.81, 130.04, 129.78, 129.62, 126.78, 125.15, 123.64, 123.43, 122.14, 118.63, 117.57, 117.23, 115.23, 114.35, 108.92, 70.60; ESI-TOF-HRMS calcd for $C_{26}H_{17}ClN_6O_3$ (*m/z*) $[M+H]^+$ 497.1129, found 497.1114.

***N*-(4-((3-azidobenzyl)oxy)-3-chlorophenyl)-6-(5-((but-3-yn-1-ylamino)methyl)furan-2-yl)quinazolin-4-amine (lapaAZY).** To a stirred solution of **31** (36.0 mg, 0.0726 mmol), AcOH (6.0 μL, 0.080 mmol), and 3-buthynylamine (7.0 μL, 0.087 mmol) in 1,2-dichloroethane (3 mL) was added $NaBH(OAc)_3$ (29.0 mg, 0.137 mmol) at 0 °C. The mixture was warmed to room temperature and further stirred for 1 h. To the mixture was added saturated aqueous $NaHCO_3$ and MeOH at 0 °C, and further stirred for 10 min. The resulting mixture was

extracted with AcOEt, dried over Na_2SO_4 and concentrated under reduced pressure. The residue was purified by silica gel column chromatography ($CHCl_3$/MeOH = 30:1) to afford the title compound (25.5 mg, 0.0464 mmol, 64%) as a yellow solid. ^{1}H-NMR (500 MHz, $CDCl_3$) δ 8.67 (s, 1H), 8.21 (s, 1H), 7.96 (s, 1H), 7.94 (d, J = 7.5 Hz, 1H), 7.83 (s, 1H), 7.82 (d, *J* = 7.5 Hz, 1H), 7.55 (dd, J = 8.5, 2.3 Hz, 1H), 7.36 (dd, J = 8.0, 8.0 Hz, 1H), 7.22 (d, J = 8.0 Hz, 1H), 7.15 (s, 1H), 6.98 (dd, J = 8.0, 1.7 Hz, 1H), 6.93 (d, J = 8.5 Hz, 1H), 6.67 (d, J = 3.0 Hz, 1H), 6.32 (d, J = 3.0 Hz, 1H), 5.09 (s, 2H), 3.90 (s, 2H), 2.86 (t, J = 6.6 Hz, 2H), 2.45 (td, J = 6.6, 2.5 Hz, 2H), 2.01 (t, J = 2.5 Hz, 1H); ^{13}C-NMR (125 MHz, $CDCl_3$) δ 157.76, 154.74, 153.66, 152.38, 151.15, 149.30, 140.56, 138.70, 132.39, 130.11, 129.20, 129.01, 128.97, 124.97, 123.60, 123.51, 122.07, 118.67, 117.64, 115.34, 114.64, 114.43, 110.21, 107.38, 82.12, 70.67, 70.12, 47.18, 45.93, 19.40; ESI-TOF-HRMS calcd for $C_{30}H_{24}ClN_7O_2$ (*m/z*) $[M+H]^+$ 550.1753, found 550.1725.

Supplementary References

Astleford, B.A., Goe, G.L., Keay, J.G., and Scriven, E.F.V. (1989). Synthesis of 1-alkyl-1,2,4-triazoles: a new one-pot regiospecific procedure. J. Org. Chem. *54*, 731–732.

Heeres, J., Backx, L.J.J., and Van Cutsem, J. (1984). Antimycotic azoles. 7. Synthesis and antifungal properties of a series of novel triazol-3-ones. J Med Chem *27*, 894–900.

Jones, J.E., Slack, J.L., Fang, P., Zhang, X., Subramanian, V., Causey, C.P., Coonrod, S.A., Guo, M., and Thompson, P.R. (2012). Synthesis and screening of a haloacetamidine containing library to identify PAD4 selective inhibitors. ACS Chem Biol *7*, 160–165.

Pace, J.R., Deberardinis, A.M., Sail, V., Tacheva-Grigorova, S.K., Chan, K.A., Tran, R., Raccuia, D.S., Wechsler-Reya, R.J., and Hadden, M.K. (2016). Repurposing the Clinically Efficacious Antifungal Agent Itraconazole as an Anticancer Chemotherapeutic. J Med Chem *59*, 3635–3649.

Shi, W., Nacev, B.A., Bhat, S., and Liu, J.O. (2010). Impact of Absolute Stereochemistry on the Antiangiogenic and Antifungal Activities of Itraconazole. ACS Med Chem Lett *1*, 155–159.

Tanoury, G.J., Hett, R., Wilkinson, H.S., Wald, S.A., and Senanayake, C.H. (2003). Total synthesis of (2R,4S,2′S,3′R)-hydroxyitraconazole: implementations of a recycle protocol and a mild and safe phase-transfer reagent for preparation of the key chiral units. Tetrahedron: Asymmetry *14*, 3487–3493.
