# Supplementary figures and images for: Image-based screen capturing misfolding status of Niemann-Pick type C1 identifies potential candidates for chaperone drugs
Source: PLoS One. 2020 Dec 14;15(12):e0243746. doi: 10.1371/journal.pone.0243746 (PMC7735562; doi:10.1371/journal.pone.0243746)

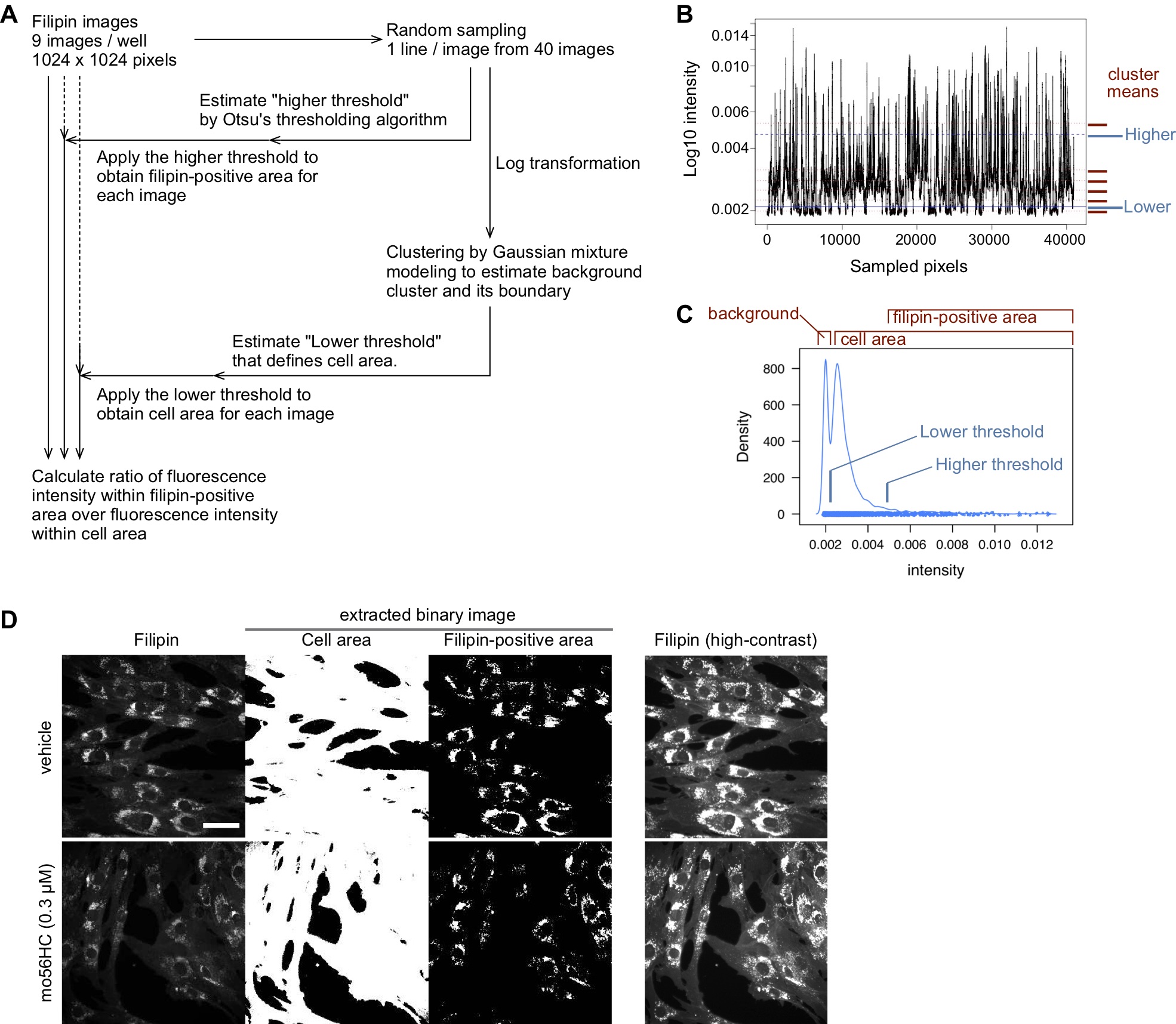

Supplement: S1 Fig — (A) Schematic workflow of the analysis of filipin-stained images. (B) A concatenated intensity profile from randomly selected images, showing the presence of high-intensity filipin-positive regions (mostly LE/L compartment), low-intensity regions originated from within-cell area, and background regions originated from areas without cells. The red lines on the right of the plot represent cluster means, and the blue lines represent the “lower threshold” and “higher threshold”. Note that image intensity in R is scaled to 0 to 1. (C) Distribution of the pixel intensities plotted in (B), along with the lower and higher thresholds, showing the presence of background clusters. (D) Representative intermediate images from the workflow, showing successful extraction of cell-covered area and filipin-positive, vesicular area. (JPG) [file pone.0243746.s002.jpg]

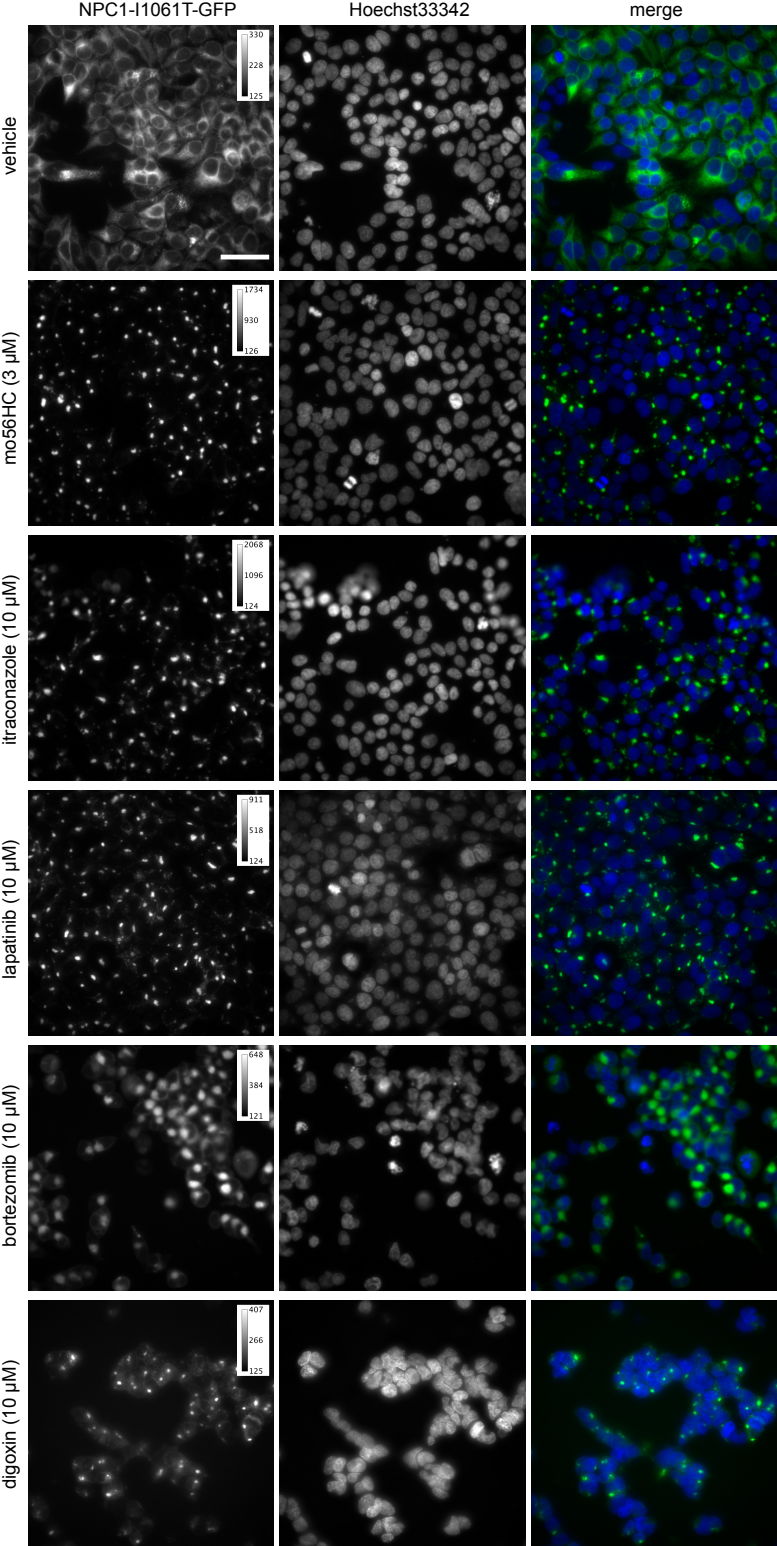

Supplement: S2 Fig — Representative screening images from cells treated with mo56HC, itraconazole, lapatinib, bortezomib, and digoxin are shown. To better visualize the subcellular localization pattern, contrast of each images was adjusted image-by-image basis. Scale bar, 50 μm. (PDF) [file pone.0243746.s003.pdf]

**A**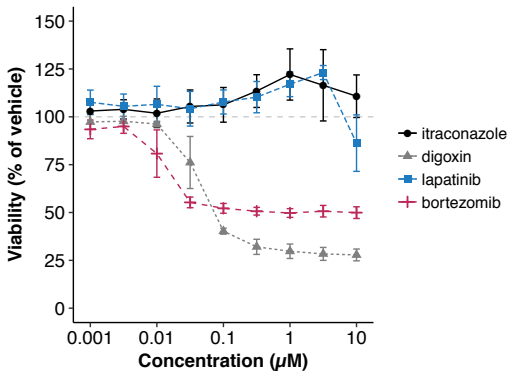**B**

|              | IC50 ( $\mu\text{M}$ ) |        |
|--------------|------------------------|--------|
|              | mean                   | SD     |
| itraconazole | > 10                   | —      |
| lapatinib    | > 10                   | —      |
| digoxin      | 0.0488                 | 0.0181 |
| bortezomib   | 0.0145                 | 0.0058 |

Supplement: S3 Fig — HEK293 cells were treated with the indicated concentrations of itraconazole, digoxin, lapatinib, and bortezomib for 20 h, and viability of the cells was assessed by alamarBlue assay. The data points represent the mean from three independent experiments each performed in triplicate, and the error bars denote standard deviations from the independent experiments. The IC50 values shown in the table were calculated from each independent experiment, and represented as mean ± SD (n = 3). (PDF) [file pone.0243746.s004.pdf]

# Conditions

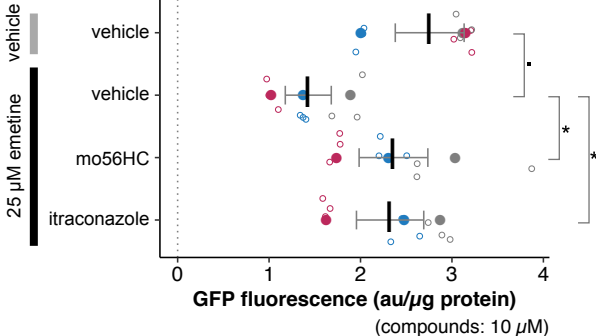

Supplement: S4 Fig — To confirm that the up-regulatory effect of itraconazole is due to reduced degradation of the NPC1-I1061T-GFP, we tested if itraconazole shows up-regulatory effect even in the presence of emetine. The cells expressing NPC1-I1061T-GFP were treated as indicated in the presence of 25 μM emetine for 18 h, and the expression level of NPC1-I1061T-GFP was assessed as previously described (GFP measurement after lysis with TNET buffer, and normalized with total protein concentration measured by BCA assay) [20]. The data were independently collected three times, and each experiment was performed in biological triplicates. The open circles represent the biological replicates and the filled circles represent their average, and the data was visualized as a SuperPlot to better represent experimental variation and reproducibility [36]. Mean and SEM of the independent experiments were shown as bold lines and error bars. Statistical significance was assessed by paired t-test with Benjamini-Hochberg p adjustment for multiple comparisons (., p<0.1; *, p<0.05). (PDF) [file pone.0243746.s005.pdf]

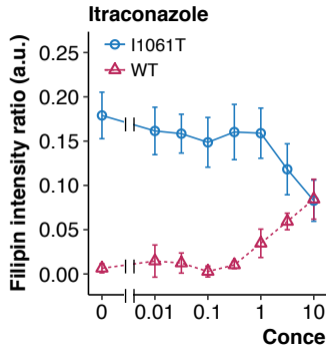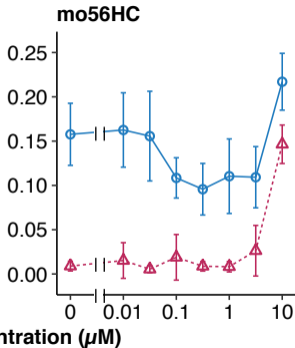

Supplement: S5 Fig — As reported previously [20], mo56HC treatment of NPC1-I1061T cells showed biphasic response, where cholesterol accumulation is alleviated at lower concentration, but higher concentration of mo56HC rather induced cholesterol accumulation, which was also observed for WT cells. In contrast, itraconazole induced cholesterol accumulation in WT cells, but slightly reduced cholesterol accumulation in I1061T cells through currently unclear mechanisms. The data represents mean ± SD of nine fields of images. (PDF) [file pone.0243746.s006.pdf]
