# Supplementary material for: Image-based screen capturing misfolding status of Niemann-Pick type C1 identifies potential candidates for chaperone drugs
Source: PLoS One. 2020 Dec 14;15(12):e0243746. doi: 10.1371/journal.pone.0243746 (PMC7735562; doi:10.1371/journal.pone.0243746)
Supplement: Attachment — Submitted filename: Plosone.pdf [file pone.0243746.s007.pdf]

Shioi et al develop an image-based screen to discover novel NPC1-I1061T chaperones. This is a timely study as chaperone-based therapeutics have become of significant interest in the Niemann-Pick C. Field. The authors use a GFP NPC1-I1061T fusion system to develop, implement a drug-repurposing screen, and interpret their positive hits. The authors obtain several promising leads and further characterize their effect of I1061T ubiquitination and function. This is exciting and novel work; however, the authors need to address the following point to confirm their hypothesis and better understand how these compounds are functioning on NPC1.

1: The bortezomid and itraconazole screening images should be included in the manuscript for comparison. Currently it is only discussed.

2: All gels need to be quantified.

3: The authors need to better establish how their lead compound itraconazole modulates I1061T. The authors indicate itraconazole reduces ubiquitination however, 4B indicates an increase in poly-ub with itraconazole?

4: Shioi nicely show itraconazole increases co-localization of NPC1-I1061T with the lysosomal compartment and later show this lysosomal form does not correct filipin. Interestingly, fig. 4B bottom suggests that itraconazole accumulates an immature glycosylated form of NPC1-I1061T. The authors indicate itraconazole is known to inhibit glycosylation however, there is an alternative interpretation of this data. Schultz et al (Nature Comm 2018) showed NPC1-I1061T is also degraded from the ER by both ERAD and ER-autophagy. They similarly found that NPC1-I1061T from ER-phagy entering the lysosome was not-functional and EndoH sensitive. It is possible that itraconazole prevents ERAD but still allows ER-phagy to occur. The authors should perform an EndoH assay to empirically determine the glycosylation state of NPC1-WT and NPC1-I1061T plus and minus itraconazole or comment on this alternative interpretation.

5: The authors should comment if there a pool of GFP-NPC1-I1061T which does not fold properly and cannot be seen in the clear-native PAGE gels.

Minor

1: Although the authors include nuclear staining to assess cell death, cell death needs to be formally assessed with dose curves of the lead compounds (at a minimum itraconazole).

2: The authors should determine if NPC1-I1061T stability is modified with itraconazole.
