# Supplementary material for: Image-based screen capturing misfolding status of Niemann-Pick type C1 identifies potential candidates for chaperone drugs
Source: PLoS One. 2020 Dec 14;15(12):e0243746. doi: 10.1371/journal.pone.0243746 (PMC7735562; doi:10.1371/journal.pone.0243746)
Supplement: Attachment — Submitted filename: 20200929_ResponseToReviewers.docx [file pone.0243746.s009.docx]

# Response to the editor’s & reviewers’ comments

## Re: Editor’s comment

Thank you for submitting your manuscript to PLOS ONE. After careful consideration, we feel that it has merit but does not fully meet PLOS ONE’s publication criteria as it currently stands. Therefore, we invite you to submit a revised version of the manuscript that addresses the points raised during the review process.
There are a few number of remaining issues that need to be addressed before we can further consider the manuscript.

We are grateful to the Editor and reviewers for taking time to evaluate our revised manuscript. We addressed the remaining issues on the molecular weight marker in the native-PAGE data and “monomer/oligomer” nomenclature of the observed NPC1-GFP species as described below.

## Re: Reviewer #1

Reviewer #1: Considering the difficulties of performing laboratory work during COVID-19, Shioi et. al. did a commendable job addressing my major concerns. The following minor points still exist:

We are really grateful to the reviewer for the kind consideration on the current situation.

1) In fig 4A the authors describe I1061T as a monomer and oligomer. While NPC1 has been reported to be a dimer, this is still controversial and the authors focus on correcting the “monomeric” NPC1. The authors should include molecular weight markers on the gel so that we can at least see if the “oligomers” represent ubiquitinated NPC1 species. To simplify the nomenclature, the authors should consider renaming the monomer and oligomer to just NPC1 and high molecular weight species.

We agree that the native oligomeric state of NPC1 remains a controversial; some reported as dimer and some as monomer.

First of all, we would like to clarify that the native PAGE protocol we used here (fluorescence-detectable, clear-native PAGE) utilizes a mixture of a mild anionic detergent and non-ionic detergent [ref 26 in the manuscript, Ihara et al., 2011 (Anal Biochem 412, 217-223)]. In contrast to the most-common blue-native PAGE, this method has been reported to give larger apparent molecular weight for membrane proteins compared to soluble proteins due to the bound detergent micelles [ref 26]. Thus, the commercially available molecular weight markers could not be used to deduce the actual size of the observed NPC1-GFP species, and indeed the “monomer” band was found near the 440 kDa marker (ferritine) and the “oligomer” band was observed slightly above the 669 kDa marker (thyroglobulin) in our preliminary experiments. Thus, to discuss the exact size of the observed species, we may need to use blue native-PAGE combined with western blotting analysis (as the blue color interferes with GFP detection), which we could not perform under the current situation.

So to clarify why we do not discuss the molecular weight of the observed species, we added a sentence *“Please note that the CN-PAGE is reported to give larger apparent molecular weight for membrane proteins compared to the soluble molecular weight markers, due to bound detergent micelles, and we could not deduce the size of the observed species [26]”.*

Regarding the second issue, “monomer/oligomer” issue, we would like to clarify that our main focus here is to show the different mobility of the NPC1-GFP bands and not to discuss the exact identity of the observed bands nor exact oligomeric status of the NPC1 GFPs. Therefore, following the suggestion by the reviewer, we renamed the “monomer/oligomer” to “faster migrating band/slower migrating species” to emphasize the electrophoretic difference rather than assuming their oligomerization state. However, we commented a possible identity of the slower migrating species as *“possibly arisen from oligomerization due to partial denaturation”* as in the following sentence: *“Considering that thermal denaturation of proteins results in a reduced monodisperse band/peak [40,41], a plausible explanation would be that the slower migrating band is possibly arisen from oligomerization due to partial denaturation and the faster/slower band ratio reflects the stability and/or folding status of WT and the I1061T mutant.”*

Accordingly, the “monomer/oligomer” in the Fig 4A and the “monomeric peak ratio”in Fig 4C were replaced with “faster migrating species / slower migrating species” and “faster band ratio”.

2) In the discussion the authors imply that since intraconazole binds a similar region as oxysterols it “establishes intraconazole as a pharmacological chaperone”. However, without proper I1061T glycosylation and correction of filipin this is not the case. The authors need to revise this statement to less definitive like they did in the abstract.

We thank the reviewer for pointing this out. The word “establish” was indeed an overstatement, without data on the correction of immature glycosylation and cholesterol accumulation. Following the reviewer’s advice, we toned down the statement to *“…, supporting our hypothesis that itraconazole acts as a pharmacological chaperone for NPC1”* as in the abstract.
